# Supplementary material for: Leukotriene receptor antagonists and eosinophilic granulomatosis with polyangiitis: a disproportionality analysis from FAERS, JADER, CVAR databases integrated with network pharmacology
Source: PLoS One. 2026 Mar 9;21(3):e0343084. doi: 10.1371/journal.pone.0343084 (PMC12970897; doi:10.1371/journal.pone.0343084)
Supplement: S1 Table — (DOCX) [file pone.0343084.s001.docx]

**S1 Table.** Checklist of READUS-PV guidelines for reporting disproportionality analyses.

| **Section and topic** | **Item #** | **Checklist item** |
| --- | --- | --- |
| **Title** |  |  |
|  | 1a | If disproportionality analyses are a prominent component of the published study, the study should be identified as a “disproportionality analysis”. The type of data and name of the database(s) should be specified. |
|  | 1b | Report the name of adverse event(s) and/or drug(s) under study, when applicable. |
| **Introduction** |  |  |
| Background | 2a | Describe the drug(s) and its utilization, the nature of the adverse event(s) under study and its frequency, and the existing knowledge on the drug-event combination. |
|  | 2b | Specify the rationale for performing the analysis, e.g., as part of routine pharmacovigilance, to investigate an overall safety profile, or to assess a pre-specified hypothesis. |
|  | 2c | Explain why ICSR databases and disproportionality analysis are suitable to fill the knowledge gap. |
| Objectives | 3 | State specific objectives, identifying the adverse event(s), the drug(s), and the reference group, including any pre-specified hypothesis, if applicable. |
| **Methods** |  |  |
| Study design | 4a | Identify the study (i.e., “disproportionality analysis”) and the type of data used (e.g., “individual case safety reports”). |
|  | 4b | Provide an outline of the entire study design, including primary and sensitivity analyses performed, and other designs such as case-by-case analysis or literature review. |
| Data description, access, and pre-processing | 5a | Specify the name of the database(s), the database(s) custodian, and the coverage. Specify the type/number of drugs included within the database and the thesaurus, taxonomies, or ontologies used for coding drugs and events. |
|  | 5b | Specify the extraction dates and describe and justify all choices used for data pre-processing, including any data transformation or exclusion, if appropriate. |
| Variables definition | 6a | Describe the study population, including any restriction. |
|  | 6b | Describe the nature and the meaning of key variables assessed in the work. |
|  | 6c | Specify and justify any grouping of drugs or events. For drugs, specify and justify whether active ingredients/trade names/salts were considered and/or the selected role. |
|  | 6d | Describe any additional data source used, the type of data, and how they interact with ICSRs. |
| Statistical methods | 7a | Present any descriptive analysis performed, specifying variables investigated, statistical tests, and significance thresholds. |
|  | 7b | Describe the measure(s) selected for the disproportionality analysis including any threshold used to identify signals of disproportionate reporting. Explain the reason for this choice if applicable. |
|  | 7c | Clearly describe any sensitivity analysis and any tool to control confounding, including any restriction, subgroup, stratification, adjustment, or interaction. |
|  | 7d | Specify the variables and methods used for the case-by-case analysis, including any algorithm or criteria used to assess causality, if performed. |
|  | 7e | Specify any statistical methods used for other data sources. |
| **Results** |  |  |
| Participants | 8a | Specify the number of individual case safety reports included at each stage, including reasons for exclusion. |
|  | 8b | Provide key demographic and clinical characteristics of cases, if possible comparing cases with any appropriate reference group. |
| Disproportionality analysis | 9 | Present all results including confidence intervals. Present also results of sensitivity analyses, if performed. |
| Case-by-case analysis | 10 | Present the case-by-case analysis of key variables. Present the causality assessment, if applicable. |
| **Discussion** |  |  |
| Key results | 11 | Discuss key results with reference to study objectives and contextualize them within the current literature and other consulted sources. Clearly discriminate between expected reactions and emerging safety signals. |
| External validity | 12a | Discuss the external validity of the results to the general population. |
|  | 12b | Discuss the potential relevance of results in clinical practice |
|  | 12c | Propose further study designs if applicable |
| Limitations | 13 | Present general limitations, making clear that disproportionality analysis alone cannot prove causation or measure incidence, and specific limitations, including confounding and reporting bias and efforts to mitigate them. |
| **Declarations** |  |  |
|  | 14a | Provide the source of funding/sponsorship and the role of the funders/sponsors for the present study and for any original study on which the present article is based. |
|  | 14b | Clearly identify potential commercial and intellectual conflicts of interest (e.g., link to any drug/event investigated, whether financial, legal action, or software used). |
|  | 14c | Declare any institutional approval needed or granted in the investigation. |
|  | 14d | Include a statement on data availability, code availability (including the version of the statistical software used), and protocol registration. |
