# Supplementary material for: Leukotriene receptor antagonists and eosinophilic granulomatosis with polyangiitis: a disproportionality analysis from FAERS, JADER, CVAR databases integrated with network pharmacology
Source: PLoS One. 2026 Mar 9;21(3):e0343084. doi: 10.1371/journal.pone.0343084 (PMC12970897; doi:10.1371/journal.pone.0343084)
Supplement: S2 Table — (DOCX) [file pone.0343084.s002.docx]

S2 Table. The generic, trade, and former names of leukotriene receptor antagonists.

|  | Montelukast | Zafirlukast | Pranlukast | Ibudilast |
| --- | --- | --- | --- | --- |
| Generic name | Montelukast | Zafirlukast | Pranlukast | Ibudilast |
| Trade name | Singulair | Accolate |  | Ketas |
| Former name | MK 0476  MK-0476  MTLU | ICI 204,219  ICI 204219  ICI-204219  Aeronix  Olmoran | ONO 1078  ONO-1078  SB 205312  ONO-RS 411  ONO-RS-411 | KC 404  KC-404  AV-411  AV411  AV 411  MN-166  MN166 |
| Japan name | プランルカスト | モンテルカスト | モンテルカスト | モンテルカスト |
