# Supplementary material for: Leukotriene receptor antagonists and eosinophilic granulomatosis with polyangiitis: a disproportionality analysis from FAERS, JADER, CVAR databases integrated with network pharmacology
Source: PLoS One. 2026 Mar 9;21(3):e0343084. doi: 10.1371/journal.pone.0343084 (PMC12970897; doi:10.1371/journal.pone.0343084)
Supplement: S3 Table — (DOCX) [file pone.0343084.s003.docx]

S3 Table. Formulas for the 4 algorithms in disproportionality analysis.

| Model | Formula | Thresholds |
| --- | --- | --- |
| ROR | $\text{ROR}\text{=}\frac{\text{a}\text{/}\text{c}}{\text{b}\text{/}\text{d}}$  $\text{SE}\text{(}\ln\text{R}\text{OR}\text{)=}\sqrt{\left( \frac{\text{1}}{\text{a}}\text{+}\frac{\text{1}}{\text{b}}\text{+}\frac{\text{1}}{\text{c}}\text{+}\frac{\text{1}}{\text{d}} \right)}$  $\text{95\%}\text{CI}\text{=}\text{e}^{\ln\text{(}\text{ROR}\text{)±1.96}\text{se}}$ | a≥3  95%CI of the ROR > 1 |
| PRR | $\text{PRR}\text{=}\frac{\text{a}\text{/(}\text{a}\text{+}\text{b}\text{)}}{\text{c}\text{/(}\text{c}\text{+}\text{d}\text{)}}$  $\text{χ}^{\text{2}}\text{=}\frac{\text{(}\text{ad}\text{−}\text{bc}\text{)}^{\text{2}}\text{(}\text{a}\text{+}\text{b}\text{+}\text{c}\text{+}\text{d}\text{)}}{\text{(}\text{a}\text{+}\text{b}\text{)(}\text{c}\text{+}\text{d}\text{)(}\text{a}\text{+}\text{c}\text{)(}\text{b}\text{+}\text{d}\text{)}}$ | a≥3  PRR > 2  *χ*2 ≥4 |
| BCPNN | $\text{IC}\text{=}\text{log}_{\text{2}} \frac{\text{p}\text{(}\text{x}\text{,}\text{y}\text{)}}{\text{p}\text{(}\text{x}\text{)}\text{p}\text{(}\text{y}\text{)}}\text{=}\text{log}_{\text{2}} \frac{\text{a}\text{(}\text{a}\text{+}\text{b}\text{+}\text{c}\text{+}\text{d}\text{)}}{\text{(}\text{a}\text{+}\text{b}\text{)(}\text{a}\text{+}\text{c}\text{)}}$  $\text{E}\text{(}\text{IC}\text{)=}\text{log}_{\text{2}} \frac{\text{(}\text{a}\text{+}\text{γ}\text{11)(}\text{a}\text{+}\text{b}\text{+}\text{c}\text{+}\text{d}\text{+}\text{α}\text{)(}\text{a}\text{+}\text{b}\text{+}\text{c}\text{+}\text{d}\text{+}\text{β}\text{)}}{\text{(}\text{a}\text{+}\text{b}\text{+}\text{c}\text{+}\text{d}\text{+}\text{γ}\text{)(}\text{a}\text{+}\text{b}\text{+}\text{α}\text{1)(}\text{a}\text{+}\text{c}\text{+}\text{β}\text{1)}}$  $\text{V}\text{(}\text{IC}\text{)=}\frac{\text{1}}{\text{(}\ln\text{2}\text{)}^{\text{2}}}\left[ \begin{aligned} \frac{\text{(}\text{a}\text{+}\text{b}\text{+}\text{c}\text{+}\text{d}\text{)−}\text{a}\text{+}\text{γ}\text{−}\text{γ}\text{11}}{\text{(}\text{a}\text{+}\text{γ}\text{11)(1+}\text{a}\text{+}\text{b}\text{+}\text{c}\text{+}\text{d}\text{+}\text{γ}\text{)}}\text{+}\frac{\text{(}\text{a}\text{+}\text{b}\text{+}\text{c}\text{+}\text{d}\text{)−(}\text{a}\text{+}\text{b}\text{)+}\text{a}\text{−}\text{α}\text{1}}{\text{(}\text{a}\text{+}\text{b}\text{+}\text{α}\text{1)(1+}\text{a}\text{+}\text{b}\text{+}\text{c}\text{+}\text{d}\text{+}\text{α}\text{)}} \\ \text{+}\frac{\text{(}\text{a}\text{+}\text{b}\text{+}\text{c}\text{+}\text{d}\text{+}\text{α}\text{)−(}\text{a}\text{+}\text{c}\text{)+}\text{β}\text{−}\text{β}\text{1}}{\text{(}\text{a}\text{+}\text{b}\text{+}\text{β}\text{1)(1+}\text{a}\text{+}\text{b}\text{+}\text{c}\text{+}\text{d}\text{+}\text{β}\text{)}} \end{aligned} \right]$  $\text{γ}\text{=}\text{γ}\text{11}\frac{\text{(}\text{a}\text{+}\text{b}\text{+}\text{c}\text{+}\text{d}\text{+}\text{α}\text{)(}\text{a}\text{+}\text{b}\text{+}\text{c}\text{+}\text{d}\text{+}\text{β}\text{)}}{\text{(}\text{a}\text{+}\text{b}\text{+}\text{α}\text{1)(}\text{a}\text{+}\text{c}\text{+}\text{β}\text{1)}}$  $\text{IC}\text{−2}\text{SD}\text{=}\text{E}\text{(}\text{IC}\text{)−2}\sqrt{\text{V}\text{(}\text{IC}\text{)}}$ | a≥3  IC025>0 |
| MGPS | $\text{EBGM}\text{=}\frac{\text{a}\text{(}\text{a}\text{+}\text{b}\text{+}\text{c}\text{+}\text{d}\text{)}}{\text{(}\text{a}\text{+}\text{c}\text{)(}\text{a}\text{+}\text{b}\text{)}}$  $\text{SE}\text{(}\ln\text{E}\text{BGM}\text{)=}\sqrt{\left( \frac{\text{1}}{\text{a}}\text{+}\frac{\text{1}}{\text{b}}\text{+}\frac{\text{1}}{\text{c}}\text{+}\frac{\text{1}}{\text{d}} \right)}$  $\text{95\%}\text{CI}\text{=}\text{e}^{\ln\text{(}\text{EBGM}\text{)±1.96}\text{se}}$ | a≥3  EBGM05>2 |

a, the number of cases with target adverse event (AE) reported with the drug of interest; b, the number of all other events reported with the drug of interest; c, the number of cases with target AE reported with the comparator; d, the number of all other events reported with a comparator; ROR, reporting odds ratio; PRR, proportional reporting ratio; BCPNN, Bayesian confidence propagation neural network; MGPS, Multi-Item Gamma Poisson Shrinker; CI, confidence interval; χ^2^: chi-squared; IC, information component; IC025, Information Component 2.5th percentile; E (IC), expected IC; V(IC), variance of IC; EBGM: Empirical Bayes Geometric Mean; EBGM05, lower limit of 95% CI of EBGM
