# Supplementary material for: Leukotriene receptor antagonists and eosinophilic granulomatosis with polyangiitis: a disproportionality analysis from FAERS, JADER, CVAR databases integrated with network pharmacology
Source: PLoS One. 2026 Mar 9;21(3):e0343084. doi: 10.1371/journal.pone.0343084 (PMC12970897; doi:10.1371/journal.pone.0343084)
Supplement: S4 Table — (DOCX) [file pone.0343084.s004.docx]

S4 Table. Related targets of LTRAs from different databases.

|  | SwissTargetPrediction  database | CTD database | Targetnet databases | Pharmmapper |
| --- | --- | --- | --- | --- |
| Montelukast | 100 | 54 | 15 | 98 |
| Zafirlukast | 100 | 44 | 25 | 96 |
| Pranlukast | 100 | 8 | 40 | 98 |
| Ibudilast | 100 | 8 | 103 | 94 |
| Total | 400 | 114 | 183 | 386 |

CTD, Comparative Toxicogenomics database; LTRAs, leukotriene receptor antagonists.
