# Supplementary figures and images for: Leukotriene receptor antagonists and eosinophilic granulomatosis with polyangiitis: a disproportionality analysis from FAERS, JADER, CVAR databases integrated with network pharmacology
Source: PLoS One. 2026 Mar 9;21(3):e0343084. doi: 10.1371/journal.pone.0343084 (PMC12970897; doi:10.1371/journal.pone.0343084)

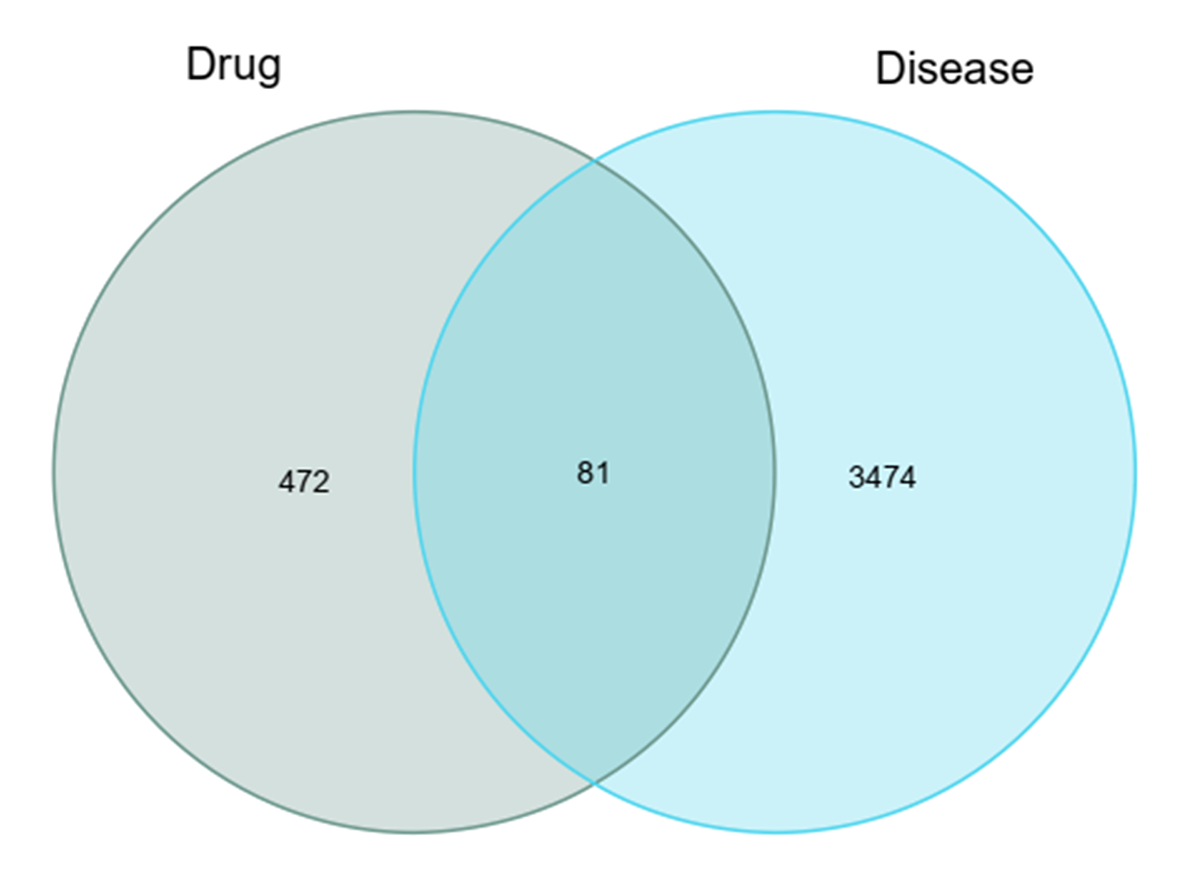

Supplement: S1 Fig — LTRAs, leukotriene receptor antagonists; EGPA, eosinophilic granulomatosis with polyangiitis. (TIF) [file pone.0343084.s005.tif]

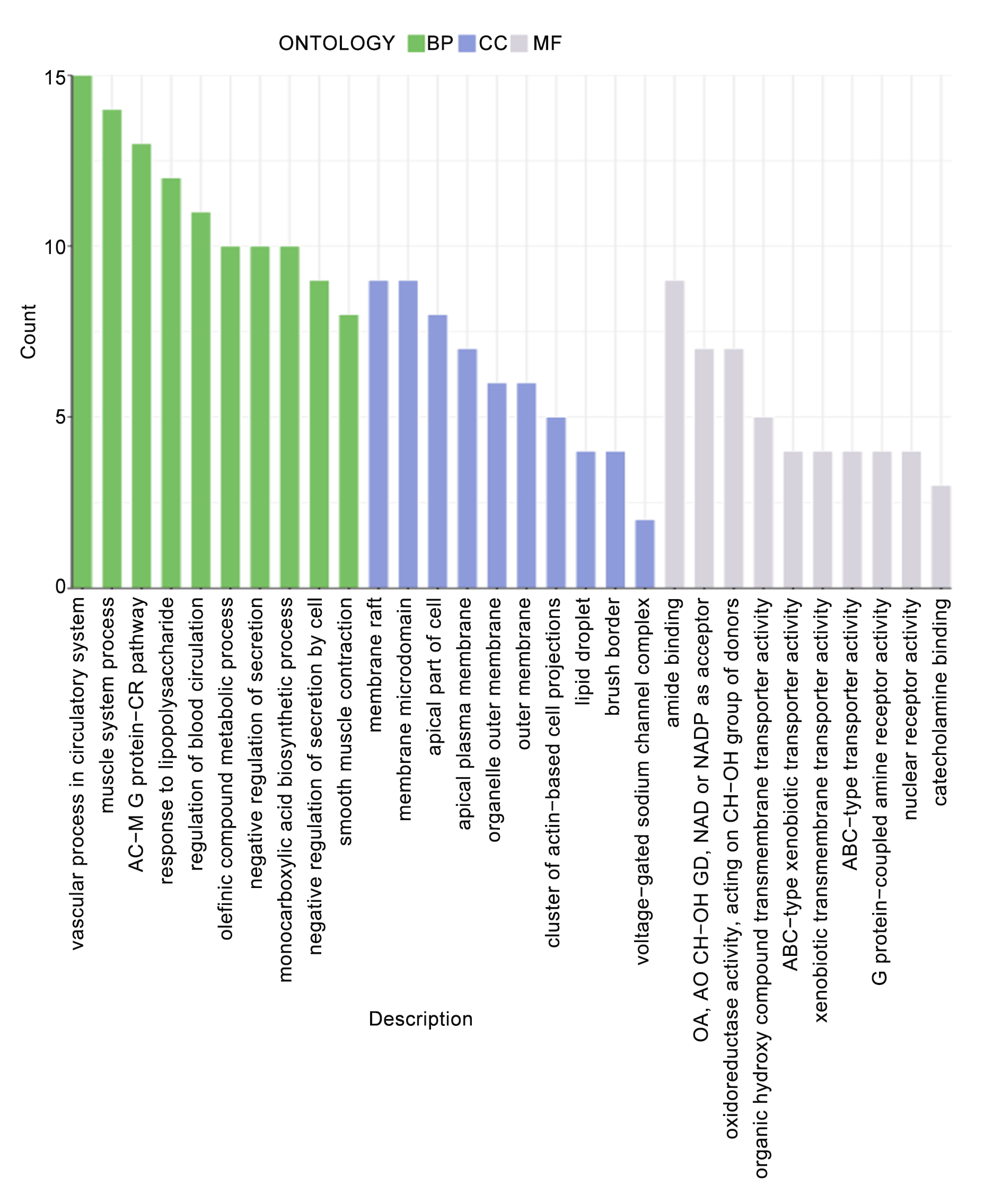

Supplement: S2 Fig — LTRAs, leukotriene receptor antagonists; EGPA, eosinophilic granulomatosis with polyangiitis; GO, Gene Ontology; CC, cellular components; BP, biological processes; MF, and molecular functions; AC − M G protein−CR pathway, adenylate cyclase−modulating G protein−coupled receptor signaling pathway; OA, AO CH − OH GD, NAD or NADP as acceptor, oxidoreductase activity, acting on the CH − OH group of donors, NAD or NADP as acceptor. (TIF) [file pone.0343084.s006.tif]
